# Reviewed article: Ribeiro LF; Moura ED; Guimarães PC; Rosa TA. Completeness and clinical-epidemiological profile of notifications of venomous animal envenomations: a descriptive analysis in the Centro-Sul region of the State of Rio de Janeiro, 2020-2023. Epidemiol Serv Saude. 2026:35;e20250541

**DOI:** 10.1590/S2237-96222026v35e20250541.c

**Published:** 2026-11-09

**Authors:** Gabriel Pavinati

**Affiliations:** 1 Universidade Estadual de Maringá, Maringá, PR, Brazil Universidade Estadual de Maringá Maringá PR Brazil

## First round comments

O manuscrito necessita de uma revisão substancial em sua apresentação em todas as seções, com ênfase nos aspectos metodológicos, que devem ser ajustados e descritos de forma mais clara e detalhada. Recomenda-se fortemente a utilização do checklist STROBE (https://www.strobe-statement.org/checklists/) como guia para garantir maior rigor e transparência na descrição metodológica.

Destaco ainda a importância de uma análise cuidadosa dos pareceres emitidos pelos revisores, assegurando que todas as solicitações sejam atendidas. Nos casos em que não for possível contemplá-las, é imprescindível que haja justificativa consistente, devidamente fundamentada na literatura pertinente.

A seguir, apresento alguns pontos críticos que requerem atenção especial, além daqueles já apontados pelos pareceristas:

- Reestruturação do resumo, com maior detalhamento da seção metodológica e apresentação dos resultados e conclusão de que se atenham aos achados da pesquisa.
- Em relação ao objetivo, apesar de ser informado que ele seria “descrever o perfil epidemiológico de acidentes causados por animais peçonhentos [...], na cidade de Três Rios/Rio de Janeiro”, no dataset disponibilizado constam casos com outras unidades notificadoras, a exemplo de Levy Gasparian, Paraíba do Sul. Como os autores justificam essa questão?
- Rever a denominação do tipo de estudo, que se enquadra como epidemiológico do tipo descritivo, e não qualitativo. Além disso, detalhar melhor o cenário da pesquisa e o período da coleta de dados; explicar o processo de seleção de dados e se houve critérios de seleção (exclusão/inclusão); de que maneira os dados foram tabulados e analisados; como foram tradados os dados ignorados, quais são os potenciais vieses da pesquisa considerando o seu caráter local (limitado a um serviço municipal) e como isso implica na interpretação dos resultados.
- Nos resultados, a descrição narrativa das tabelas está prolixa, atentar-se a destacar os achados mais relevantes e dispensar a descrição integral dos dados já apresentados na tabela.
- Remover todas as afirmações que não são resultados da pesquisa e que não foram corroboradas pela literatura científica (ex: “O presente trabalho compreende o primeiro estudo epidemiológico sobre acidentes causados por animais peçonhentos notificados em Três Rios”).
- Casos particulares não carecem de descrição detalha, uma vez que o objetivo do estudo não é esse, como no parágrafo: “Foi observado um caso envolvendo picada de lacraia e outro caso associado a envenenamento por lagartas. No primeiro, a paciente, uma mulher de 64 anos [...]”
- Na discussão, os autores não abordaram a possível influência do cenário sanitário atípico (pandemia da covid-19), na ocorrência dos acidentes e na notificação dos casos estudados. Incluir
- O último paragrafo da discussão, tipicamente a conclusão, precisa responder diretamente ao objetivo da pesquisa, nesse caso, de maneira quantitativa, inclusive. Rever
- Rever a qualidade e atualizar das referencias utilizadas, uma vez que mais de 20 das 30 referencias são anteriores a cinco anos e/ou literatura cinzenta. Reavaliar pertinência de cada uma e optar por sua atualização.

Em consonância aos pareceristas, recomendo: que o manuscrito seja aprimorado em sua estrutura geral, garantindo que o título descreva de forma clara e envolvente o tema central do estudo, que o resumo seja independente, conciso e completo, incluindo objetivos, métodos, resultados e conclusões. Sugiro ainda que a introdução apresente o problema, o conhecimento já estabelecido, a lacuna existente e objetivos consistentes; que os métodos sejam detalhados quanto ao desenho, seleção dos dados, variáveis, e estratégias de análise; e que os resultados tragam dados bem-organizados, caracterização da amostra. Na discussão, é importante resumir e interpretar os achados, relacioná-los à literatura, reconhecer limitações e propor aplicações práticas e pesquisas futuras. Por fim, recomendo atenção especial às referências, que devem ser atualizadas e formatadas conforme o periódico.

Recommendation: Major revision 

## Second round comments

Pendências

1. Incluir a menção ao checklist STROBE no método, bem como sua citação e referência.
2. A descrição no subitem “tamanho do estudo” repete informações do subitem “participantes”. Rever
3. Nos resultados, remover juízo de valor das frases: Foram utilizadas 251 ampolas de soro antiescorpiônico, administradas não apenas em casos graves e moderados, [conforme orientações do Ministério da Saúde], mas também em 18 dos aplicado a três pacientes.

Recommendation: Minor revision 

